# Seize the Data: A User-Friendly GUI for High-Resolution Analysis of Seizure Dynamics in HD-MEA Recordings

**DOI:** 10.1523/ENEURO.0432-25.2026

**Published:** 2026-06-03

**Authors:** Melissa L. Blotter, Jacob H. Norby, Jacob Cahoon, Katelyn C. Forbes, Logan A. Stephens, Micah R. Shepherd, R. Ryley Parrish

**Affiliations:** ^1^ Neuroscience Center, Brigham Young University; ^2^ Departments of Cell Biology and Physiology, Brigham Young University; ^3^ Computer Science, Brigham Young University; ^4^ Physics and Astronomy, Brigham Young University

**Keywords:** epilepsy, graphical user interface, status epilepticus

## Abstract

High-density multielectrode arrays (HD-MEAs) generate large, complex datasets that are challenging to efficiently manage and analyze with existing tools, especially in open-source environments. To address this, we developed the BYU Seizure and Analytics Tool (YSA), an open-source graphical user interface built in Python and C++ for efficient analysis and visualization of HD-MEA recordings. The YSA features raster plots, automated discharge detection and tracking, downsampling, playback, and export functions, enabling streamlined workflows for large-scale neural data. We demonstrate the utility of the tool in the context of seizure and status epilepticus-like activity, highlighting how the YSA facilitates rapid exploration of the spatiotemporal dynamics in brain networks. This platform provides an accessible and practical solution for HD-MEA data analysis, supporting a range of neuroscience applications.

## Significance Statement

HD-MEAs generate rich spatiotemporal datasets well suited for studying complex brain dynamics such as seizure activity. However, the size and complexity of these recordings can hinder analysis and interpretation. Existing analysis tools are often proprietary, expensive and prohibit user modification. We present the BYU Seizure and Analytics Tool (YSA), an open-source graphical user interface for intuitive visualization and exploration of MEA data. YSA enables users to navigate activity across the entire brain slice, identify relevant patterns, and export selected data for targeted analyses. By making high-density seizure data more accessible and actionable, YSA streamlines analysis pipelines and supports deeper insight into spatiotemporal neural dynamics, including seizure initiation and propagation in models of status epilepticus and pharmacoresistant epilepsy.

## Introduction

The ability to monitor neural activity across thousands of electrodes simultaneously has transformed the landscape of neural recording, thanks to advances in high-density multielectrode array (HD-MEA) systems (van Vliet et al., 2007; Maccione et al., 2010; Blotter et al., 2024). These systems are used in neuroscience and other fields to study network-level phenomena with exceptional spatiotemporal precision (Liu et al., 2012). However, the volume and complexity of data generated by HD-MEAs present major challenges for data handling, analysis, and visualization, particularly for researchers lacking access to proprietary or specialized software (Obien et al., 2014; Muller et al., 2015).

To address this need, we developed the BYU Seizure and Analytics Tool (YSA), an open-source graphical user interface (GUI) built in Python and C++. YSA is designed specifically to streamline the analysis of HD-MEA recordings, integrating essential features such as customizable raster plots, automated discharge detection, real-time playback, and easy data export into a single, user-friendly platform. Its flexible design enables researchers to quickly explore spatiotemporal patterns in large-scale electrophysiological data in a fully customizable and open-access environment while remaining accessible to users with limited programming experience.

Several open-source tools for MEA analysis are currently available, including platforms like SpyKING CIRCUS (Yger et al., 2018), MEAnalyzer (Dastgheyb et al., 2020), NeuroScope (Hazan et al., 2006), and the Xenon LFP Analysis Platform (Mahadevan et al., 2022). While these tools offer powerful capabilities, many are optimized for single-unit or burst-level dynamics and are less suited for visualizing and tracking complex spatiotemporal seizure activity. For example, the Xenon LFP Analysis Platform provides basic signal viewing and spectral features but lacks the customizable discharge-level tracking, interactive visualization, and modular flexibility needed for modern HD-MEA seizure analysis. Additionally, many of these platforms are not easily adaptable to evolving recording technologies or require substantial scripting experience. In contrast, YSA offers a real-time, user-friendly interface that enables detailed per-discharge analysis, synchronized raster and trace visualization, seamless data export, and compatibility with current and future MEA systems. Its modular, open-source design supports easy integration of user-defined routines, making it uniquely suited for high-throughput and translational seizure research.

To showcase the analytical capabilities of YSA in a real-world context, we applied it to ex vivo recordings from an acute seizure model. This seizure model presents complex, dynamic patterns of seizure-like and status epilepticus (SE)-like activity (Walther et al., 1986; Voss and Sleigh, 2010; Reddy and Kuruba, 2013; Codadu et al., 2019a,b), making it an ideal test case for evaluating the tool's ability to detect, visualize, and quantify large-scale network phenomena. While our study is grounded in epilepsy research, the tool is broadly applicable to any experimental paradigm involving HD-MEA data. User-specific routines can be easily added to the GUI for custom applications. YSA offers a robust, extensible solution for high-throughput neural data analysis and contributes to the growing ecosystem of open-source tools in neuroscience.

## Materials and Methods

### Ethical approval

All animal procedures were conducted in accordance with protocols approved by the Brigham Young University Institutional Animal Care and Use Committee and complied with the guidelines set forth by the BYU Office of Laboratory Animal Welfare.

### Animals and brain slice preparation

**Figure 1.**
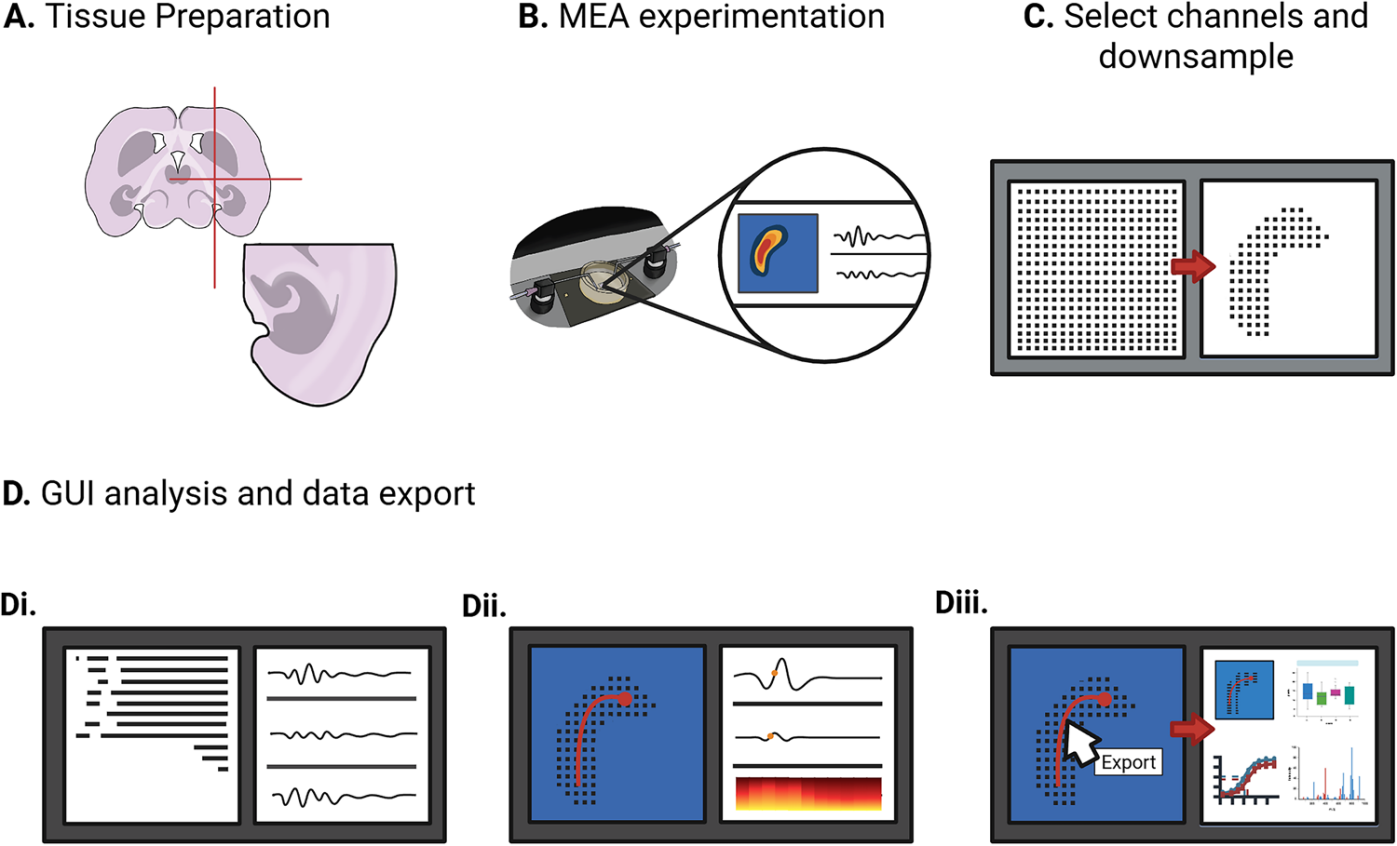
Workflow of experimentation and analysis. ***A***, Diagram of an acute mouse horizontal brain slice that includes the hippocampus, neocortex, and entorhinal cortex. ***B***, Brain slice placed onto the MEA where seizure activity is induced and recorded. ***C***, Raw recordings are downsampled spatially and temporally to facilitate fast analysis. ***D***, The processed data are loaded into the YSA, which (***Di***) generates raster plots, (***Dii***) enables visualization of individual discharges and spectrograms, and (***Diii***) allows for easy export of data for downstream analysis. Created in BioRender. https://BioRender.com/c22quko.

Acute horizontal brain slices were prepared from male and female C57BL/6 mice aged Postnatal Day (P)17–P35. Mice were anesthetized with isoflurane and killed via decapitation, in accordance with approved institutional animal care protocols. Brains were rapidly extracted and immersed in a cold cutting solution containing the following (in mM): 126 NaCl, 3.5 KCl, 1.26 NaH_2_PO_4_, 26 NaHCO_3_, 10 glucose (C_6_H_12_O_6_), and 3 MgCl_2_. Brains were sectioned into 350-µm-thick horizontal slices using a Leica VT1200S Vibratome. Slices were subdissected to isolate the neocortex, entorhinal cortex, hippocampus, and subiculum (Fig. 1*A*).

Following dissection, slices were transferred to a holding chamber containing artificial cerebrospinal fluid (aCSF) composed of the following (in mM): 126 NaCl, 3.5 KCl, 1.26 NaH_2_PO_4_, 26 NaHCO_3_, 10 glucose, 1 MgCl_2_, and 2 CaCl_2_ for a 1-hour incubation. All solutions were continuously bubbled with carbogen (95% O_2_/5% CO_2_) to maintain proper oxygenation and pH.

### Electrophysiological recordings on HD-MEA

After incubation, slices were placed on a 3Brain Accura HD-MEA chip containing 4,096 electrodes spaced 60 µm apart, arranged in a 3.8 × 3.8 mm grid (Fig. 1*B*). Recordings were performed using the 3Brain BioCAM DupleX system. Prior to induction of epileptiform activity, slices were perfused with standard aCSF (composition described above) for 10 min to allow for recovery and equilibration. Slices were then perfused in a low Mg^2+^ aCSF solution composed of the following (in mM): 126 NaCl, 3.5 KCl, 1.26 NaH_2_PO_4_, 26 NaHCO_3_, 10 glucose, and 2 CaCl_2_, to induce epileptiform activity. The solution was continuously perfused at an inflow rate of 5.0 ml/min and an outflow rate of 7.0 ml/min and maintained at physiological temperature (35–37°C) using an in-line heating system. Each recording session lasted up to 3 h and was conducted as previously described (Blotter et al., 2024).

Images of each brain slice on the 3Brain Accura chip were captured using a Dino-Lite Edge digital microscope to ensure precise localization of recording channels relative to the targeted brain regions. Data were sampled at 2,000 Hz with a 1.0 Hz high-pass filter applied and no low-pass filter.

### Data preprocessing

Recorded data were downsampled both spatially and temporally using publicly available custom code designed for the 3Brain recordings used in this study (Lab, 2025; available at https://shorturl.at/vLEgN). The code aligns an image of the brain slice with the MEA electrode array, enabling mapping of anatomical regions to electrodes and spatial downsampling via manual channel selection. This allows isolation of region-specific channels for independent analysis and summary metric generation. Spatial downsampling removes irrelevant channels, including nontissue regions, perfusion-related artifacts, and unstable electrodes (Fig. 1*C*).

Temporally, data were downsampled from 2,000 to 100 Hz to remove extraneous frequencies and reduce computational load, though YSA supports higher sampling rates (≥2,000 Hz) with increased processing time. YSA is optimized for local field potential (LFP) analysis rather than single-unit spike detection; thus, higher sampling rates (3–5 kHz) are not required. The 1.0 Hz high-pass filter applied by the hardware reduces baseline drift, and post hoc downsampling via scipy.signal.resample mitigates aliasing by removing frequencies above the Nyquist limit prior to reconstruction.

Data must be converted to HDF5 format for YSA compatibility; example code and file structure are provided in the ReadTheDocs guide (Lab, 2025). This conversion also enables analysis outside proprietary software environments.

A total of 19 slices were analyzed, yielding 54 self-limiting seizure-like events (SLSLEs) and 15 SE events. Functional channel counts varied by slice due to placement. During analysis, YSA applies an automatic signal-to-noise ratio (SNR) threshold to exclude noisy or nonfunctional channels, and all remaining channels within the selected slice area are included in event detection, visualization, and quantification.

### Custom GUI for seizure detection

#### Overview and usage

**Figure 2.**
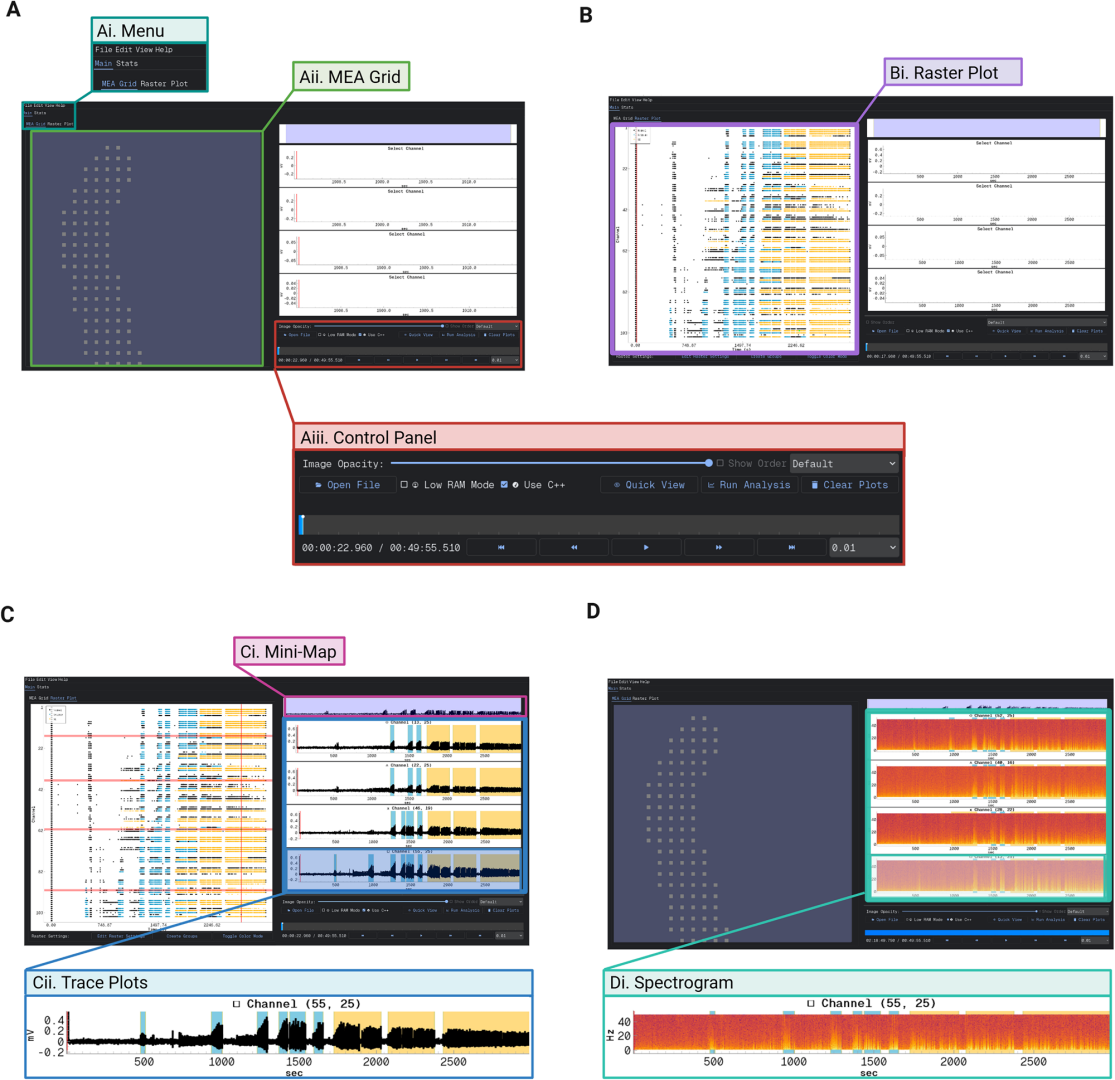
YSA overview. ***A***, Main interface of the YSA tool. ***Ai***, The full GUI view with the menu panel highlighted (turquoise box), which allows users to navigate between tabs. ***Aii***, The MEA Grid tab (green box) showing the electrode layout and signal overview across the array. ***Aiii***, The control panel (red box), which includes options for uploading recordings, adjusting playback speed, and navigating the dataset. ***B***, Raster plot view. ***Bi***, The raster tab (purple box) displays network-wide spike activity, with automated annotations for seizure-like events (light blue) and SE-like activity (yellow). ***C***, Trace plot view. ***Ci***, The minimap timeline (pink box) provides a compressed view of the full recording for navigation. ***Cii***, Individual trace plots (blue box) display voltage traces from selected electrodes. One trace is enlarged to show axis labels and temporal detail. ***D***, Spectrogram view. ***Di***, Example spectrograms from individual electrodes (light green box), showing frequency content over time. A combined trace-spectrogram view is enlarged to display axes and resolution. Created in BioRender. https://BioRender.com/7e9puvu.

The downsampled data were imported into YSA (Figs. 1*D*, 2*A*). Upon loading, users are presented with a control panel for importing data, adjusting parameters, and navigating the interface (Fig. 2*A*). A sidebar menu (turquoise) allows access to various tabs, including a visualization of the MEA grid with active electrode positions (Fig. 2*A*). Using the overlaid brain slice image, users can define distinct groups within the grid to differentiate between brain regions. Users can highlight up to four individual channels to display the signals with quick zoom-in and zoom-out and pan options. A list of default parameters and a guide for parameter tuning can be found in the ReadTheDocs guide (Lab, 2025).

**Figure 3.**
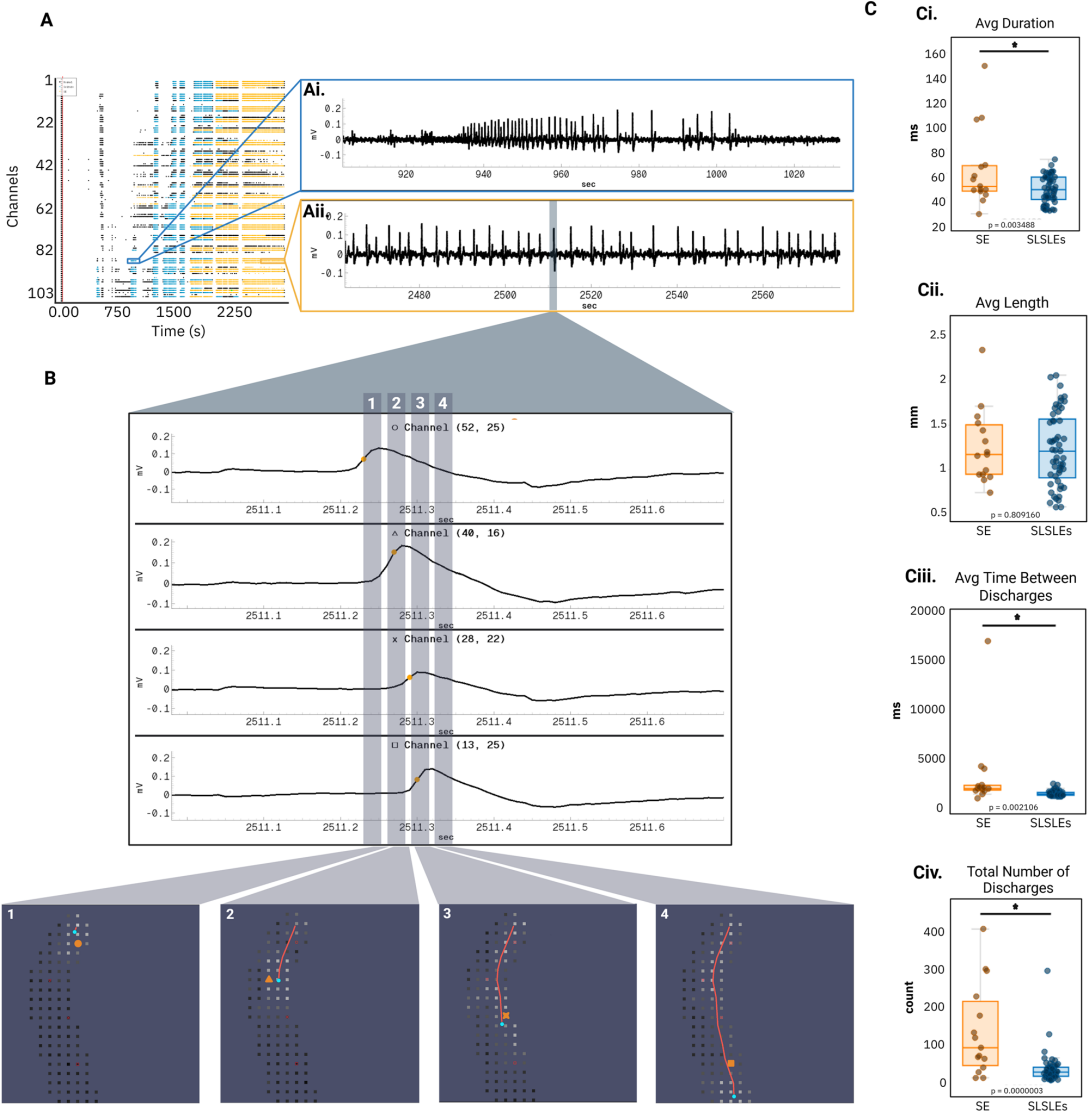
Seizure and SE discharge analysis using the YSA. ***A***, Example raster plot recorded from a brain slice. ***Ai***, Representative trace of a SLSLE. ***Aii***, Representative trace of SE. ***B***, Zoomed-in view of an individual discharge event. Panels 1–4 highlight distinct phases of a discharge, with corresponding electrode maps showing the spatial propagation across the brain slice. ***C***, Quantitative comparisons between SE and SLSLE events, (***Ci***) average duration (*p* = 0.0035), (***Cii***) average path length (*p* = 0.81), (***Ciii***) average time between discharges (*p* = 0.0021), and (***Civ***) total average number of discharges in an SE or SLSLE event (*p* = 0.000003). Each scatter point represents the averaged discharge values for a single event (SE, *n* = 15; SLSLE, *n* = 53). All statistical tests were two-tailed *t* tests. Created in BioRender. https://BioRender.com/ya87tc8.

Switching to the raster plot tab displays spike activity across all electrodes over time, with detected SLSLEs (light blue) and SE events (yellow) overlaid automatically (Figs. 2*B*, 3*A*). Additionally, the raster plot can be ordered in terms of groups that the user has defined as previously mentioned. Naturally, detection routines can be updated (with only small amounts of coding) to fit the user's specific needs. Raster plots can also be displayed alongside trace plots for any selected channels, allowing close inspection of raw signals during identified events (Fig. 2*C*). A minimap panel (pink) provides an overview of the entire recording timeline, indicating the currently displayed window.

In addition, users can view time–frequency representations of activity via per-electrode spectrograms (Fig. 2*D*), enabling frequency-domain inspection of discharges and their evolution across the array. Spectrogram parameters can be modified within the edit settings menu.

#### Seizure detection algorithm

YSA can be used to identify regions that exhibit SLSLEs and SE-like activity using a seizure detection algorithm. This algorithm is performed on each channel individually in three steps: First, it identifies a reference or baseline section of a 2 min period with no significant activity spikes. Next, it identifies voltage outliers that deviate by 3 standard deviations (SD) from the baseline. For each of these, the algorithm checks whether the variance in that window also deviates by 3 SD from the reference variance. The resulting set of points is defined as discharges. Discharges occurring within 2 s of each other are grouped together in the same event. These events are then categorized by their duration: those lasting longer than 10 s are classified as SLSLEs, those shorter than 10 s are discarded, and those lasting longer than 5 min are classified as SE (Dulla et al., 2018). Improved detection routines or other custom algorithms can be easily added to the source code to replace the default algorithm.

The YSA detection algorithm was benchmarked against other published open-source seizure–detection algorithms (Table 1; Mahadevan et al., 2022; Vermeulen et al., 2025) that were also designed using LFP rodent model data. The respective algorithms were run on the same set of 872 single channel LFP signals that were each ∼1 h long and randomly selected from 19 unique MEA recordings. As different algorithms may subdivide seizure types, the benchmarking method is only considered if an event was detected or not. The performance was scored on a by-sample basis against a human annotated “ground-truth” set of identified seizure-like events for each signal. On a sample-by-sample basis, the rate of true-positive, true-negative, false-positive, and false-negative samples was recorded and compiled to generate the data displayed in Table 1. The timing of running each detection algorithm was determined for each signal using the built-in MATLAB and Python timing functions, without parallelization. Furthermore, the timing analysis was carried out on an 8 GB RAM, Apple M1 processor, and the timing of the same algorithms will likely differ on systems with greater RAM and processing capability; however, the comparison between algorithms should remain true.

**Table 1.** Benchmarking of SLE detection algorithms

Algorithm	Avg. speed (±2 SD; s)	Accuracy	Precision	Recall	Specificity	*F*1
Xenon LFP	2.33 ± 2.52	0.929	0.962	0.951	0.822	0.957
Vermeulen	5.58 ± 1.42	0.864	0.903	0.935	0.521	0.919
YSA	2.12 ± 1.82	0.889	0.932	0.934	0.676	0.933

Algorithm detection benchmarking was performed on 872 ∼1-h-long individual channel recordings from 19 unique MEA recordings. The performance of algorithms was compared on a by-sample basis to a “ground-truth” of events for each signal as determined beforehand by human reviewers. Accuracy is a measure of overall performance; Accuracy = (TP + TN)/(TP + FP + TN + FN). Precision is a measure of how well the algorithm evaluates positive samples, Precision = TP/(TP + FP). Recall is a measure of how well the model detects all positive instances, Recall = TP/(TP + FN). Specificity is a measure of the algorithm's ability to correctly detect negative samples, Specificity = TN/(TN + FP). *F*1 provides a balances measure of precision and recall, *F*1 = (2 × Precision × Recall)/(Precision + Recall). TP = # of true-positive samples detected by an algorithm, TN = # of true-negative samples detected by an algorithm, FP = # of false-positive samples detected by an algorithm, FN = # of false-negative samples detected by an algorithm.

### Tracking and mapping discharges

The YSA GUI includes a peak detection module to identify all discharge peaks across electrodes. With user-set parameters for minimum peak threshold (in SD from the mean), minimum distance between peaks (in samples), and SNR, peaks are identified in each channel. For each peak, the preceding time window is analyzed to locate the point with the largest voltage difference, marking this as the discharge onset. This approach ensures alignment between detected discharges and their representation on the false color map. For our analysis, peak detection was initialized using a threshold of 4 SD above the mean, a minimum interpeak distance of 30 samples, a maximum detection window of 20 samples, a bin size of 0.0133, and a SNR threshold of 35. These parameters were refined on a per-recording basis as needed to ensure accurate identification of physiologically relevant discharges while minimizing noise detection.

Once peaks are identified, YSA tracks their propagation across the slice using the DBSCAN algorithm (from the scikit-learn Python package, which also has tunable parameters in the settings menu) to place the center of the discharge at each time point as the discharge travels. The default DBSCAN parameters will likely need to be adjusted based on the individual noise level of each recording. Epsilon is a measure of how far the DBSCAN algorithm should look to cluster (at least a minimum of the electrode-to-electrode distance), and MinPts is the minimum number of points required to make a cluster. Refining these depends on the spatial resolution and downsampling of the data being analyzed. For our analysis, DBSCAN was initialized with epsilon = 4.8 and MinPts = 4, and parameters were adjusted per recording to optimize clustering of propagating discharges while minimizing inclusion of noise. Additionally, it allows users to mark and monitor the electrodes involved in discharge initiation, enabling analysis of spatial discharge patterns and timing intervals between events. Using the overlaid slice image, users can correlate these electrodes to areas of the brain slice.

### Discharge feature quantification

For each detected discharge, YSA computes multiple temporal and spatial features. Discharge duration was defined as the time between the first and last detected discharge onsets within a given discharge. Interdischarge interval was defined as the time between the onset of consecutive discharges within the same event. The total number of discharges per event was defined as the count of individual discharges detected within a continuous SLSLE or SE episode.

To quantify spatial propagation, YSA computes the propagation path length for each discharge. During tracking, DBSCAN clustering is applied within successive temporal bins to identify groups of active electrodes, and the spatial centroid of each cluster is computed. These centroids are linked across time to form a discharge trajectory. The propagation path length is then calculated as the sum of the Euclidean distances between consecutive centroids along this trajectory. A discharge is considered complete after three consecutive time bins without newly detected clusters. The default bin size is 0.0133 s, although this parameter is user-adjustable within the YSA interface.

### Statistical analyses

YSA performs signal processing, epileptiform event detection, feature extraction, and visualization at the level of individual recordings. Extracted features and event metrics are exported as structured .csv tables to enable flexible downstream statistical analysis across multiple recordings and experimental groups in external environments (e.g., MATLAB, Python, or R). This design allows users to aggregate data from many slices or animals and apply customized large-scale analyses, including population dynamics, synchrony metrics, and functional connectivity.

In this study, statistical analyses were performed on .csv outputs from YSA using custom MATLAB code and automated where possible. Data from two groups were analyzed using two-tailed *t* tests for all plots. Figures were generated in MATLAB and BioRender.

### Software accessibility

The YSA was developed in Python (v3.10.9) and is compatible with Windows and macOS. Source code and user documentation are available at Lab (2025). The software relies on standard Python libraries including [PyQt5, pyqtgraph, scipy, pandas, scikit-learn]. A detailed README file provides setup instructions and usage guidelines, as well as an example data file (Lab, 2025).

## Results

Using YSA's automated detection and tracking features, we identified and visualized individual discharges within both SLSLE and SE events. The tool enables spatial tracking of each discharge across electrodes (Fig. 3*B*), revealing dynamic propagation patterns over time. These visualizations support detailed spatiotemporal examination of event evolution within and across brain regions.

In comparison to other similar established detection algorithms, the built-in YSA detection algorithm performs similarly on standard detection metrics while also prioritizing speed (Table 1; Mahadevan et al., 2022; Vermeulen et al., 2025). The YSA detection method demonstrated an accuracy of 0.889, comparable to the 0.929 and 0.864 of the Xenon LFP and Vermuelen methods (Table 1). Furthermore, the YSA algorithm exhibited a 0.933 *F*1-score, also comparable to the 0.957 and 0.919 achieved by the other algorithms (Table 1). Lastly, the YSA algorithm completed detection of hour-long signals (at 100 Hz) with an average speed of 2.12 ± 1.82 s, slightly faster than the Xenon algorithm (2.33 ± 2.52) and notably faster than the Vermuelen algorithm (5.58 ± 1.42 s). As different algorithms may subdivide seizure types, the benchmarking method only considered if an event was detected or not, and the performance was scored on a by-sample basis against a human annotated “ground-truth” set of identified seizure-like events for each signal. While this provides a standard comparison between algorithms, it does omit the more specialized function of some algorithms, including the YSA algorithm. To our knowledge, YSA is currently the only open-source detection method that classifies SE in addition to standard SLSLEs.

YSA also quantified key metrics for each detected discharge, including average duration, propagation path length, interdischarge intervals, and total number of discharges per event (see Materials and Methods; Fig. 3*C*). As a demonstration of the tool's analytic capabilities, we compared these features between SLSLE and SE events. SE events exhibited significantly longer discharge durations than SLSLE (Fig. 3*Ci*). Interestingly, the average discharge length (i.e., spatial extent) did not differ significantly between states, suggesting that while SE discharges last longer in time, they do not necessarily engage in a broader spatial area (Fig. 3*Cii*). Interdischarge intervals were also longer in SE (Fig. 3*Ciii*), and SE events contained a greater number of total discharges (Fig. 3*Civ*). These results reflect and expand on differences between pharmacoresistant and pharmacosensitive seizure states and illustrate how YSA facilitates high-resolution, event-level comparisons (Sanya, 2004; Barker-Haliski et al., 2017). These comparisons reveal distinct dynamic features of pharmacoresistant SE, such as increased temporal persistence without broader spatial recruitment. This insight may point to evolving mechanisms of local circuit hyperexcitability, offering clues into the network-level transitions that underline treatment-resistant seizure progression.

**Figure 4.**
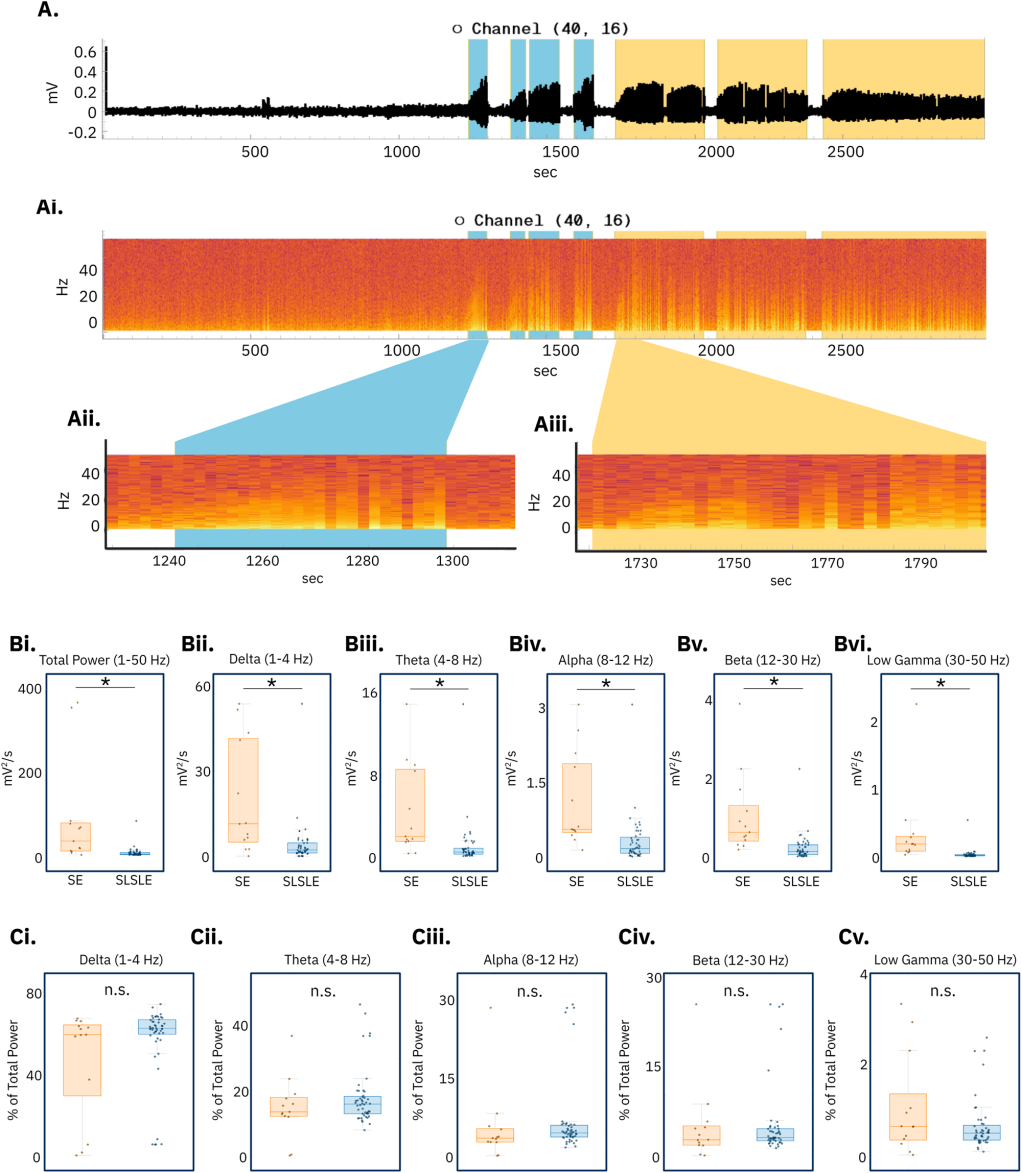
Spectral analysis of SLSLE and SE events. ***A***, An example of an electrophysiology trace from the YSA. ***Ai***, An example of a spectrogram generated by the YSA overlapping the selected trace. ***Aii***, A zoomed-in view of the spectrogram during an SLSLE. ***Aiii***, A zoomed-in view of the spectrogram during an SE event. ***B***, The power, normalized to time, of SE events was significantly greater than the power of SLSLEs in (***Bi***) total band power 1–50 Hz (*p* = 5.1 × 10^−5^), (***Bii***) delta power 1–4 Hz (*p* = 4.1 × 10^−5^), (***Biii***) theta power 4–8 Hz (*p* = 2.4 × 10^−4^), (***Biv***) alpha power 8–12 Hz (*p* = 8.5 × 10^−5^), (***Bv***) beta power 12–30 Hz (*p* = 1.8 × 10^−5^), and (***Bvi***) low gamma power 30–50 Hz (*p* = 5.2 × 10^−4^). ***C***, The percentage of total power (normalized to time) of SE events was not significantly different than the power of SLSLEs in (***Ci***) delta power 1–4 Hz (*p* = 0.12), (***Cii***) theta power 4–8 Hz (*p* = 0.28), (***Ciii***) alpha power 8–12 Hz (*p* = 0.55), (***Civ***) beta power 12–30 Hz (*p* = 0.88), and (***Cv***) low gamma power 30–50 Hz (*p* = 0.071). All statistical tests were two-tailed *t* tests. Created in BioRender. https://BioRender.com/w2vl6t4.

YSA spectral analysis allows togglable spectrograms and export of event spectra for downstream analysis (Fig. 4*A*). Analysis of absolute spectral power demonstrated that SE events were significantly more powerful than SLSLE events across the full frequency range (1–50 Hz; *p* = 5.1 × 10^−5^) and within standard frequency bands (Fig. 4*Bi*–*vi*; delta, 1–4 Hz; *p* = 4.1 × 10^−5^; theta, 4–8 Hz; *p* = 2.4 × 10^−4^; alpha, 8–12 Hz; *p* = 8.5 × 10^−5^; beta, 12–30 Hz; *p* = 1.8 × 10^−5^; and low gamma, 30–50 Hz; *p* = 5.2 × 10^−4^). In contrast, the proportional contribution of each frequency band to the total power (percent of total) was not significantly different between SE and SLSLE events (Fig. 4*Ci*–*v*; delta, 1–4 Hz; *p* = 0.12; theta, 4−8 Hz; *p* = 0.28; alpha, 8–12 Hz; *p* = 0.55; beta, 12–30 Hz; *p* = 0.88; and low gamma, 30–50 Hz; *p* = 0.071). These results indicate that SE events are associated with a globally heightened state of network excitability rather than a shift in the relative distribution of power across frequency bands.

Together, these analyses demonstrate the ability of YSA to extract, visualize, and quantify both time- and frequency-domain features of HD-MEA recordings with minimal manual processing. By streamlining this process, YSA provides a robust platform for identifying subtle electrophysiological differences in seizure dynamics across experimental conditions.

## Discussion

Current MEA analysis software, while powerful, often comes with steep learning curves, limited flexibility for examining electrophysiological traces at high temporal resolution, poor adaptability to evolving recording technologies, and cost barriers (Obien et al., 2014; Muller et al., 2015; Liu et al., 2024). Many existing tools are optimized for spike and burst detection, making them less suited for tracking large-scale LFP activity during ictal or other network-wide events (Hennig et al., 2019; Dastgheyb et al., 2020). To overcome these limitations, we developed the YSA, a user-friendly, open-access GUI that combines intuitive design with robust analytical features. YSA requires no complex setup, is compatible with future recording advancements, and is ready to use upon download, enabling advanced analysis without specialized computational training.

While our demonstration used data from a 3Brain recording system (Blotter et al., 2024), the YSA can support data from any MEA recording system. Supporting documentation (Lab, 2025) provides code to convert 3Brain output files (.brw) into the universal YSA format (.h5) along with data structure guidelines so users of other MEA platforms can easily reformat their recordings for use in YSA. Existing 3Brain analysis tools are propriety and require additional licenses. YSA provides a viable alternative for researchers that allow them to modify and implement custom analyses to data collected across MEA systems.

To showcase YSA, we applied it to a well-established acute ex vivo seizure model. This model provides rich, dynamic spatiotemporal data, offering an ideal test case for evaluating the tool's analytical capabilities. While not the primary focus of this paper, our analysis illustrates how YSA enables rapid visualization, annotation, and quantification of seizure-like and SE-like events on a per-discharge basis, often difficult with traditional software.

The YSA automatically generates raster plots and extracts electrophysiological metrics commonly used in seizure analysis (Figs. 2*B*, 3*A*; Bradley et al., 2018; Fan et al., 2019; Matsuda et al., 2022). Raster plots provide insights into synchronous discharges and propagation trends (Wagner et al., 2015; Diamond et al., 2024). YSA enhances this capability by integrating synchronized trace views, enabling both broad and granular analysis of seizure dynamics. Users can track individual discharges, precisely measuring duration, speed, and spatial spread across the electrode array (Fig. 3), helping identify patterns that differentiate seizure-like states from sustained SE-like activity. These features minimize the need for custom scripting and make rigorous analysis accessible to users with varied technical backgrounds.

The YSA also supports seamless data export, simplifying integration with other analysis pipelines or inclusion in publications and presentations. By combining both standard and advanced features in a single package, YSA enhances efficiency and accessibility in HD-MEA data analysis, particularly for complex seizure dynamics.

In our demonstration, YSA enabled high-resolution tracking of individual discharges across the electrode grid, revealing distinct spatiotemporal patterns associated with SE-like activity. For example, discharges during SE had significantly longer average durations than those in SLSLEs, consistent with the sustained nature of SE (Fig. 3*Ci*; Treiman et al., 1990; Karunakaran et al., 2012). Interestingly, average discharge length (i.e., spatial extent) did not differ significantly between states, suggesting SE discharges persist longer in time without broader spatial engagement (Fig. 3*Cii*). The average time between discharges was also significantly longer in SE (Fig. 3*Ciii*). Additionally, the total number of discharges was significantly greater during SE than SLSLEs, consistent with the extended duration of SE episodes (Fig. 3*Civ*; Drislane et al., 2009; Trinka et al., 2015; Johnson and Kaplan, 2020). Together, these findings illustrate how YSA enables detection of subtle dynamic features often missed with existing software. The ability to precisely track discharge duration, frequency, and spatial spread in real time allows researchers to distinguish between SLSLEs and more prolonged, pharmacoresistant SE-like activity. By enabling high-throughput quantification of these dynamics, YSA facilitates more nuanced interpretations of network state transitions, insights that could ultimately inform both basic research and preclinical therapeutic screening.

On a broader scale, YSA's export functions enabled spectral analysis of SLSLE and SE events, showing that while SE events exhibited significantly higher absolute power across all frequency bands, the relative power distribution remained similar. These results, again, shown as a demonstration of tool capabilities, suggest that SE events reflect a sustained and elevated level of excitability rather than a fundamentally different spectral composition (Holtkamp et al., 2011; Jiruska et al., 2013; Amengual-Gual et al., 2019; Niquet et al., 2019). This kind of insight, extracted quickly from large datasets, exemplifies how YSA can accelerate data interpretation. Notably, trends toward elevated absolute power in low-frequency bands (delta, theta) have been associated with impaired inhibition and disrupted network oscillations, features typical of pharmacoresistant seizure states (Walker, 2008; Avoli et al., 2016; Toutant et al., 2024).

Finally, YSA successfully detected transitions from SLSLEs to SE, capturing dynamic shifts in network state that align with previous findings on SE progression (Fig. 2*Bi*; Anderson et al., 1986; Burman et al., 2019; Codadu et al., 2019a; Trevelyan et al., 2023). While not intended as a biological discovery, this capacity highlights YSA's value in identifying real-time network transitions, making it a valuable tool for researchers studying seizure generalization and pharmacoresistant SE models.

We anticipate that this platform will support future studies aimed at uncovering mechanisms of seizure evolution and drug resistance while facilitating reproducible, high-throughput analysis of HD-MEA data (Thouta et al., 2022; Goodchild et al., 2024). Beyond seizure research, the modular and data-agnostic structure of YSA makes it broadly applicable across neuroscience and related fields. For example, the ability to visualize and quantify spatiotemporal patterns of electrical activity at the single-discharge level may prove valuable in studying network oscillations, plasticity, injury responses, or neurodevelopmental dynamics. Furthermore, because the YSA framework is customizable and compatible with a variety of HD-MEA systems, it can also be adapted for use in cardiac electrophysiology to track propagation of arrhythmic events or response to pharmacological interventions. While compatible with other HD-MEA systems, including the clinically approved Utah Array (refer to the ReadTheDocs guide; Lab, 2025), YSA does not currently support grid dimensions exceeding 64 × 64; however, this limitation could be removed in future versions.

Importantly, by enabling researchers to rapidly extract interpretable data from large-scale recordings without specialized coding knowledge, YSA lowers the barrier to entry for electrophysiological analysis. This accessibility positions it as a valuable tool for preclinical screening and drug discovery pipelines, where efficiency and reproducibility are essential. We believe that YSA not only enhances the interpretability of complex neural datasets but also expands the experimental possibilities available to researchers across a wide range of disciplines.

## Data Availability

The data and code that support the findings of this study are available from the corresponding author upon request.

## Synthesis

Reviewing Editor: Timothy Jarome, Virginia Polytechnic Institute and State University Decisions are customarily a result of the Reviewing Editor and the peer reviewers coming together and discussing their recommendations until a consensus is reached. When revisions are invited, a fact-based synthesis statement explaining their decision and outlining what is needed to prepare a revision will be listed below. The following reviewer(s) agreed to reveal their identity: NONE.

The manuscript is improved but several concerns need addressed

1. The manuscript describes spatiotemporal analysis across brain slices but does not clearly state whether the toolbox enables comparisons of seizure dynamics across distinct anatomical regions (e.g., hippocampus vs. neocortex). Given that slices include multiple regions (hippocampus, entorhinal cortex, neocortex), authors should clarify the following: Does YSA support region-wise segmentation and comparison? Can users define specific anatomical masks? Are summary metrics (e.g., discharge rates, propagation speed) exportable per region? Authors should clearly state whether such cross-regional comparisons are supported or must be performed downstream after export.

2. Authors describe discharge tracking using DBSCAN clustering and centroid trajectories but it remains unclear whether this can be used to extract structured propagation pathways along anatomical circuits (e.g., longitudinal hippocampal axis, entorhinal-hippocampal loops). Specifically: Can YSA quantify directionality of propagation? Can propagation be analyzed along known anatomical axes? This is particularly important given the relevance of propagation patterns in epilepsy.

3. Filtering (line 142): It is unclear what the rationale is for "1.0 Hz high-pass filter applied and no low pass filter." for LFPs, you need low-pass filtering, as high-frequency noise may distort signals. Further, data are later downsampled to 100 Hz, so low-pass filtering is required to avoid aliasing.

4. Line 150: "downsampling spatially excluded common HD MEA artifacts such as perfusion bubbles, non-tissue regions, and unstable channels". Unclear how these artifacts were detected and eliminated.

5. Line 194: "it identifies voltage outliers that deviate by 3 standard deviations (SD) from the baseline." Is the SD a user-adjustable parameter? If so, how should it be chosen? Ideally, one should plot signal voltages as a histogram and fit the noise using Gaussian assumptions.

6. Provide a table or separate section to list recommended default parameters (or range of parameter settings). If possible, clarify the tolerance to deviations from these defaults.

7. It was not clear whether the full experimental data analyzed (9 slices, 54 SLSLEs, 15 SE events) in this work was made publicly available for replication.

## Author Response

Reviewer Comment 1:

The manuscript describes spatiotemporal analysis across brain slices but does not clearly state whether the toolbox enables comparisons of seizure dynamics across distinct anatomical regions (e.g., hippocampus vs. neocortex). Given that slices include multiple regions (hippocampus, entorhinal cortex, neocortex), authors should clarify the following: Does YSA support region-wise segmentation and comparison? Can users define specific anatomical masks? Are summary metrics (e.g., discharge rates, propagation speed) exportable per region? Authors should clearly state whether such cross-regional comparisons are supported or must be performed downstream after export.

Response:

We have revised the manuscript to explicitly describe YSA's capability for user-defined grouping of channels corresponding to anatomical regions, facilitated through alignment with overlaid slice images (Lines: 174 - 176, 243 - 244).

YSA allows users to define region-specific groups (e.g., hippocampus, entorhinal cortex, neocortex), which are preserved in visualizations such as raster plots and individual trace displays. This enables qualitative cross-regional comparison of seizure dynamics within the interface, as channels are organized according to user-defined groups.

At present, summary metrics (e.g., discharge rate, propagation characteristics) are exported at the level of the full recording. Region-specific quantitative comparisons can be made by exporting subsets of channels corresponding to individual regions (e.g., by generating region-restricted files through additional downsampling or channel selection). Further statistical analysis, including formal cross-regional comparisons, is therefore conducted downstream following export (Lines: 150 - 151).

Reviewer Comment 2:

Authors describe discharge tracking using DBSCAN clustering and centroid trajectories but it remains unclear whether this can be used to extract structured propagation pathways along anatomical circuits (e.g., longitudinal hippocampal axis, entorhinal-hippocampal loops). Specifically: Can YSA quantify directionality of propagation? Can propagation be analyzed along known anatomical axes? This is particularly important given the relevance of propagation patterns in epilepsy.

Response:

The overlaid slice image enables users to visualize the anatomical location and spread of discharges across the tissue. To analyze propagation along a specific anatomical axis, users can spatially downsample the data to include only channels corresponding to the region of interest and perform analyses on that subset. (Lines 149 - 151).

In addition, YSA includes a "spread lines" feature that overlays directional arrows based on centroid trajectories derived from DBSCAN clustering, providing a visual representation of propagation direction across the slice. This feature is described in the read the docs documentation.

Reviewer Comment 3:

Filtering (line 142): It is unclear what the rationale is for "1.0 Hz high-pass filter applied and no low pass filter." for LFPs, you need low-pass filtering, as high-frequency noise may distort signals. Further, data are later downsampled to 100 Hz, so low-pass filtering is required to avoid aliasing.

Response:

We appreciate the reviewer's concern regarding filtering and potential aliasing. The downsampling to 100 Hz is performed post hoc using the scipy.signal.resample function, which applies an FFT-based approach that inherently removes frequency components above the new Nyquist frequency prior to reconstruction of the downsampled signal. As such, aliasing is mitigated during the resampling process (see https://docs.scipy.org/doc/scipy/reference/generated/scipy.signal.resample.html for documentation). (Lines: 156 - 159) The 1.0 Hz high-pass filter is applied at the hardware level to remove slow baseline drift and transient offsets inherent to MEA recordings (Lines: 156 - 157). While we did not apply an additional explicit low-pass filter in our preprocessing pipeline, the acquisition system includes a built-in anti-aliasing filter; however, the specific characteristics of this filter have not been publicly disclosed by the manufacturer (3Brain). It is expected that other data collection systems would have similar filters as part of their data acquisition hardware. If they did not, low- or high-pass filtering would need to be performed outside of YSA.

We chose our resample method to allow flexible (non-integer) downsampling. We note that alternative approaches, such as scipy.signal.decimate, which perform integer downsampling with an explicit low-pass filter, could be used if stricter control over filter characteristics is desired.

Reviewer Comment 4:

Line 150: "downsampling spatially excluded common HD MEA artifacts such as perfusion bubbles, non-tissue regions, and unstable channels". Unclear how these artifacts were detected and eliminated.

Response:

We note that spatial downsampling is guided by alignment of an image of the brain slice with the MEA electrode array, as described in the Methods (Lines 140-142). We have now explicitly reiterated this point in the section describing spatial downsampling (Lines 148-152).

Using this alignment, channels corresponding to non-tissue regions (e.g., areas outside the slice or affected by perfusion bubbles) are manually excluded based on their spatial location relative to the imaged tissue. In addition, unstable or noisy channels are further identified and excluded based on signal quality criteria, as described in the SNR-based filtering section (Lines 165-167).

Reviewer Comment 5:

Line 194: "it identifies voltage outliers that deviate by 3 standard deviations (SD) from the baseline." Is the SD a user-adjustable parameter? If so, how should it be chosen? Ideally, one should plot signal voltages as a histogram and fit the noise using Gaussian assumptions.

Response:

The standard deviation (SD) threshold used for voltage outlier detection is user-adjustable within the code, although it is not currently exposed through a graphical interface. In our implementation, we selected a threshold of 3 SD, corresponding to approximately 99% confidence under a Gaussian noise assumption; however, this parameter can be modified by the user depending on the desired sensitivity and characteristics of their data.

We agree that visualization of the signal distribution is valuable for parameter selection. Accordingly, we include a histogram of signal voltages with an overlaid normal distribution for the reviewer (attached below) to illustrate the underlying noise characteristics and support this choice. Users may use such visualizations with their own data to refine threshold selection for their specific application.

Finally, as YSA is open-source, the underlying detection algorithm can be readily modified or replaced to accommodate alternative approaches tailored to different experimental needs.

Reviewer Comment 6:

Provide a table or separate section to list recommended default parameters (or range of parameter settings). If possible, clarify the tolerance to deviations from these defaults.

Response:

We thank the reviewer for this suggestion. We have now included a table of recommended default parameters (Table 1) in the documentation ("Read the Docs"), which summarizes the values used in our analyses, and we cite this in the text (Lines: 177 - 178).

We note that optimal parameter selection is dataset-dependent, particularly given variability in signal quality and experimental conditions. Accordingly, these defaults are intended as practical starting points, and all parameters can be readily adjusted by the user to evaluate sensitivity and determine the most appropriate settings for a given dataset.

Table 1.

Reviewer Comment 7:

It was not clear whether the full experimental data analyzed (9 slices, 54 SLSLEs, 15 SE events) in this work was made publicly available for replication.

Response:

We thank the reviewer for raising this important point. The full experimental dataset analyzed in this study (9 slices, 54 SLSLEs, 15 SE events) is available upon reasonable request to the authors. We have clarified this in the revised manuscript (Lines: 420 - 422).
